# Direct contact between *Plasmodium falciparum* and human B-cells in a novel co-culture increases parasite growth and affects B-cell growth

**DOI:** 10.1186/s12936-021-03831-x

**Published:** 2021-07-05

**Authors:** Sreenivasulu B. Reddy, Noemi Nagy, Caroline Rönnberg, Francesca Chiodi, Allan Lugaajju, Frank Heuts, Laszlo Szekely, Mats Wahlgren, Kristina E. M. Persson

**Affiliations:** 1 Microbiology, Tumor and Cell Biology, Karolinska Institutet, Stockholm, Sweden; 2 Department of Clinical Microbiology, Karolinska University Hospital, Huddinge, Stockholm, Sweden; 3 Makerere University, Kampala, Uganda; 4 Department of Laboratory Medicine, Skåne University Hospital, Lund University, Lund, Sweden

**Keywords:** Malaria, *Plasmodium falciparum*, B-cell, Human, Culture

## Abstract

**Background:**

*Plasmodium falciparum* parasites cause malaria and co-exist in humans together with B-cells for long periods of time. Immunity is only achieved after repeated exposure. There has been a lack of methods to mimic the in vivo co-occurrence, where cells and parasites can be grown together for many days, and it has been difficult with long time in vitro studies.

**Methods and results:**

A new method for growing *P. falciparum* in 5% CO_2_ with a specially formulated culture medium is described. This knowledge was used to establish the co-culture of live *P. falciparum* together with human B-cells in vitro for 10 days. The presence of B-cells clearly enhanced parasite growth, but less so when Transwell inserts were used (not allowing passage of cells or merozoites), showing that direct contact is advantageous. B-cells also proliferated more in presence of parasites. Symbiotic parasitic growth was verified using CESS cell-line and it showed similar results, indicating that B-cells are indeed the cells responsible for the effect. In malaria endemic areas, people often have increased levels of atypical memory B-cells in the blood, and in this assay it was demonstrated that when parasites were present there was an increase in the proportion of CD19 + CD20 + CD27 − FCRL4 + B-cells, and a contraction of classical memory B-cells. This effect was most clearly seen when direct contact between B-cells and parasites was allowed.

**Conclusions:**

These results demonstrate that *P. falciparum* and B-cells undoubtedly can affect each other when allowed to multiply together, which is valuable information for future vaccine studies.

## Background

Malaria infection by *Plasmodium falciparum* is a major global killer [1], but attempts to formulate effective vaccines have proven challenging due to the poor development of host immunity. Protective immunity capable of controlling parasite growth develops only after repeated infections over a number of years and can be lost rapidly in the absence of antigenic stimulation [2–9]. The development of effective protective immunity against the asexual blood stages of malaria is known to be via both cellular and humoral effector mechanisms [2, 10–12].

*Plasmodium falciparum*-infected erythrocytes have the potential to directly interact with B-cells in different anatomical sites and induce B-cell proliferation and differentiation into antibody secreting cells. Antibodies have been shown to be crucial components of naturally acquired protective immunity against blood stage malaria, and were early shown to reduce parasitaemia and clear clinical symptoms [11]. However, a number of studies indicate that the development of B-cell memory to malaria is defective or suboptimal [5, 13–15] and the chronic antigenic activation of B-cells within the context of repeated *P. falciparum* infections may lead to abnormalities in B-cell development and trafficking [16]. Studies have shown that endemic settings result in an increased number of non-responsive “exhausted” B-cells, having reduced capacity to differentiate into antibody-secreting cells [14, 16, 17].

However, the exact mechanisms by which parasites affect B-cell homeostasis have not been well understood. To acquire protective immunity by means of vaccination, it needs to be understood how a *P. falciparum* infection modulates the dynamics of the B-cell homeostasis. However, there is no *P. falciparum* specific animal model and there are no in vitro methods available which could mimic the in vivo interactions. HIV infection, which like malaria is a chronic condition, results in changes in the B-cell compartment [18, 19] with an increased number of “exhausted” B-cells [20, 21]. These CD27^−^ atypical memory B-cells, which express inhibitory Fc-receptor-like-4 (FCRL4)/FCRL5 have been proposed to have a reduced capacity to differentiate into antibody-secreting cells [17].

Due to the lack of *P. falciparum* specific in vivo models, in vitro screening assays like invasion inhibition assays and anti-malarial drug screening assays form an essential part of anti-malarial therapeutic and vaccine development studies. The availability of novel in vitro tools may eventually provide valuable tools to facilitate anti-malarial research in the fields of vaccine development.

A basic requirement for studies of live *P. falciparum* together with other cells is growth in vitro. Traditionally, the “old-fashioned” candle-jar method is used [22], or a special low-level (around 1–3%) oxygen gas is flooded into sealed boxes placed in a 37 °C incubator, with or without shaking [23]. B-cells, on the other hand, have often been grown using incubators with 5% CO_2_ (in air which contains 21% oxygen), which are available in most laboratories. To be able to study the effect that *P. falciparum* (life cycle of 48 h) and human B-cells have on each other in detail, it is necessary to simulate the situation in the human body where both cells coexist for long periods of time. To study this interaction, this paper describes a novel in vitro co-culture method where *P. falciparum* are grown together with naïve B-cells for ten days and it was shown that parasites and B-cells have major effects on each other when allowed to multiply together, which is important knowledge for further vaccine studies. No one has cultured these cells together successfully for a long period of time, and when employing this method, it was found that parasites and B-cells in direct contact affected each other in numerous ways. Most strikingly, there was an increase in levels of parasites, and a clear expansion of B-cells. By using transwell culture plates, it was shown that this is partly, but not only, due to direct contact between B-cells and infected red blood cells (iRBC). Minimal characterization of these co-cultures was also attempted to assess the mutual impact of the culture components.

## Methods

For an overview of the co-culture process, see Table 1.

**Table 1:** Overview of 10 day co-culture of human B-cells and *P. falciparum*

Day -4	Isolate B-cells from PBMC using CD19 + Dynabeads
Day -3	Detatch B-cells from Dynabeads
	Irradiate CD40L + mouse fibroblasts
	Put B-cells together with irradiated CD40L + cells for activation
Day 0	Put activated B-cells and *P. falciparum* together in co-culture
Day 2–9	Daily monitoring of the co-culture and counting of parasitaemia by microscopy
	Media change with/without addition of cytokines and recomb CD40L on alternate days. Addition of fresh RBC if needed
Day 10	Flow cytometry of B-cells

### CD40L expressing cells

A CD40L-expressing mouse fibrosarcoma cell line (a kind gift from Prof. Eva Klein, MTC, Karolinska Institutet, Sweden) was grown in Iscove’s Modified Dulbecco’s Medium (IMDM HyClone Thermo Scientific, Vermont, USA) with 10% heat-inactivated foetal bovine serum (FBS), 100 U/mL penicillin and 100 µg/mL streptomycin (both from Gibco, Fisher Scientific). The cells were passaged every 4th day at 1:5. Cultures were regularly tested for the absence of Mycoplasma. Cells were detached using Versen (Phosphate Buffered Saline + EDTA 0.2 g/l, in house), spun down at 1500 rpm for 5 min and resuspended. The cells were irradiated at 15,000 rad to stop proliferation, washed, and placed in 12- or 24-well plates (TPP, Techno Plastic Products AG, Switzerland) together with B-cells as described below.

### Isolation and activation of CD19 + B-cells

Human B-cells were isolated from peripheral blood mononuclear cells (PBMCs) of anonymous healthy Swedish blood donors using CD19 + Dynabeads (Invitrogen, Massachusetts, USA). Blood samples were obtained from the Blood Bank in Stockholm. After detachment from the beads the following day, the B-cells were added to the plates with CD40L-expressing fibrosarcoma cells at a 3:1 ratio in IMDM + 10% FBS, 50 ng/mL IL4, 100 units/mL IL2, and incubated in 5% CO_2_ at 37 °C for 72 h. Instead of purified B-cells, in some experiments, the cell line CESS was used, which is a lymphoblastoid cell line (LCL) (a kind gift from Prof. Eva Klein). Activated B-cells were collected from the plate by washing with IMDM (the fibrosarcoma cells adhered to the plate) until most of the B-cells were recovered. Collected B-cells were washed once with IMDM, counted and resuspended in co-culture medium (90% IMDM, 5% Human AB + serum from healthy Stockholm blood donors, 5% FBS, 100 U/mL penicillin and 100 µg/mL streptomycin). As an alternative to IMDM, RPMI 1640 (Gibco, Fisher Scientific) and DMEM (Gibco, Fisher Scientific) were also tested.

### Growth of *Plasmodium falciparum*

*Plasmodium falciparum* (FCR3S1.2, FCR3S1.6 and 3D7) was grown as described, using sorbitol for synchronization [24]. 0 + RBC were obtained from The Blood bank in Stockholm.

### Co-culture of *Plasmodium falciparum* and activated B-cells

1.0 × 10^5^ activated B-cells/mL were placed in TPP plates together with iRBC at 1% haematocrit with a starting parasitaemia of 0.01% in co-culture medium (described above). This was incubated at 37 °C with 5% CO_2_ in air. Co-cultures were also performed using transwell plates (Corning Incorporated COSTAR, 0.4 µm or 3 µm pore size), where B-cells were placed in the receiver wells, and parasite-infected RBCs were placed in the inserts. 0.4 µm pores did not allow passage of merozoites or whole cells.

During the co-culture experiments, media changes were performed on alternating days at the beginning of the co-culture and daily from day 7. When 24-well plates were used, 950 µL of culture supernatant was replaced by 1.0 mL fresh co-culture media (double this volume was added to 12-well plates). Recombinant CD40L (1.0 µg/mL) (PeproTech, Sweden) and IL4 (50 ng/mL) was added at the start of the co-culture and then at every second media change. From day 7, 100 units/mL IL2 was also added at every second media change. Co-cultures with purified B-cells were maintained for 10 days. The concentration of parasites was determined every day (same time every day) using light microscopy and acridine orange staining. Whenever concentrations exceeded 1.0–1.5% in at least one well, 5–10 µL of non-infected fresh 0 + RBCs was added (equally in all wells, irrespective of the concentration of parasites in the other wells) to keep the parasite levels under control.

To assess the B-cell enumeration and estimate the number of B-cells, counting was performed in a Bürker chamber and the concentration of cells was also measured in the Clinical Chemistry hospital routine laboratory (where a flow cytometry-based method was used).

### Controls

Instead of parasites, 2 µM CpG (Hycult Biotech, Pennsylvania, USA) was added to selected wells as a control. To exclude presence of Epstein-Barr Virus (EBV) in the cultures, analysis of EBV was performed in the clinical routine laboratories of Smittskyddsinstitutet (Solna, Sweden) using real-time PCR. The viability of B-cells was ensured using a 1:1 dilution of the cell suspension with filtered 0.4% trypan blue (Sigma-Aldrich, Sweden) with 5 mM NaN_3_ in PBS pH 7.2. Using the haemocytometer chamber, viable cells were counted.

### Using CFDA-stained RBC

To make it possible to use RBC of different blood groups/origins in the co-cultures, CFDA labelled RBCs were tested and the Vybrant carboxyfluorescein diacetate succinimidyl ester (CFDA-SE) Cell Tracer Kit (Thermo Fisher Scientific) was used to label RBCs. The stock solution of CFDA-SE was dissolved in anhydrous DMSO prior to use. 15 µL of packed RBCs were suspended in 30 ml PBS pH 7.4 + 2 mM glucose containing 0.2 mM CFDA-SE and incubated for 5 min at 37 °C. The labelling reaction was stopped by adding 15 ml of IMDM + 10% FBS for 5 min. Finally, the cells were washed three times with PBS-glucose and resuspended in IMDM + 10% FBS and subsequently co-incubated with activated B-cells.

### Visualization of co-cultures by microscopy

B-cell and parasite co-cultures were visualized not only by bright field microcopy, but also after staining with acridine orange. Also, Confocal microscopy was performed as described [25] using the dye OFB (Medelixir AB, Stockholm, Sweden). Image capture was performed using excitation at 405, 488 and 630 nm.

### Characterization of co-cultures

#### Flow cytometry analysis of B-cells

All samples were treated with red blood cell lysis buffer pH 7.3 (155 mM NH_4_Cl, 10 mM KHCO_3_, 0.1 mM EDTA pH 7.2–7.4) for 10 min on ice. The cells were spun down at 1500 rpm for 5 min at 4 °C. After the supernatant was removed, the cells were washed three times with IMDM. The resulting B-cells were transferred into a V-bottom plate (Nunc, New York, USA) and aliquoted into unstained controls, single-colour controls, fluorescence minus one (FMO) controls and test samples. Cells were kept on ice at all times. After washing with flow cytometry staining buffer (PBS + 0.5% bovine serum albumin, GE Healthcare, Illinois, USA, + 2 mM EDTA, Sigma, Sweden), cell pellets in individual wells were resuspended in 100 µL flow cytometry staining buffer. 1 μL of Fc block (CD16/CD32 mAb, BD Biosciences, New Jersey, USA) was added to each sample, mixed gently and incubated on ice for 5 min. Cells were stained using CD19, CD20, CD27 (BD Biosciences) and FCRL4 (Biolegend, California, USA) for 30 min in the dark on ice and washed twice with 200 μL of ice-cold staining buffer by centrifugation at 1500 rpm for 5 min. In some experiments, CD3 (BD Biosciences) was added as a control to ensure the purity of B-cell cultures.

Cells were transferred into labelled precipitin tubes (BD Falcon) and kept in the dark on ice until analysis in a Beckman Coulter Cyan flow cytometer. Acquisition and compensation settings were determined based on FMO controls. The flow cytometry data were analysed using FlowJo (TreeStar Inc, Oregon, USA) software.

#### Human cytokine ELISA array

Multi-Analyte ELISArray (Qiagen) was used for the simultaneous detection of different/most common cytokines/chemokines in co-culture supernatants using conventional sandwich-based enzyme-linked immunosorbent assay (ELISA) technique. The 96-well ELISA microplate had been coated with a panel of target-specific capture antibodies, one in each eight-well strip allowing qualitative, relative profiling results from up to six samples. Each kit also included the corresponding detection antibodies, Antigen Standards, and a complete set of reagents. Using this kit, cell culture supernatants were assessed for the presence of cytokines IL-1α, IL-1β IL-2, IL-4, IL-5, IL-6, IL-8, IL-10, IL-12α, IL-13, IL-17α, GM-CSF and TNF-1β.

### Statistics

When culture conditions were compared, all conditions were compared to parasites without any other cells present, using *t*-test. When percentages of cells were compared using flow cytometry, one-way ANOVA was used to compare the different groups of cells to each other.

## Results

### Preparation of B-cells

As a first step in performing co-cultures of human B-cells and *P. falciparum*, CD19 + B-cells were isolated from PBMCs and the B-cells were used directly after isolation without prior activation. When these isolated B-cells were co-cultured together with parasites, between 50 and 70% of the B-cells died within 6 h (as tested with trypan blue, results from three different experiments). Because there was a need to find a way to co-culture parasites with B-cells on a more long-term basis, only activated B-cells were subsequently used in the assays. For the initial activation of B-cells, CD40L-expressing cells were used which resulted in much debris when the cells were maintained throughout the co-culture. Instead, whole cells were added for only three days of pre-activation of the B-cells, and then switched to using soluble CD40L (see Table 1).

### Co-culture of *Plasmodium falciparum* and B-cells

Different starting concentrations of parasites were tested when added to the B-cells, and when the concentration of parasites was below 0.1%, the parasites grew well for several days, when it was 0.5%, almost no parasites were observed after 96 h, and when the starting concentration was above 1%, no live parasites were observed after two parasite cycles (overgrowth and culture crash). Attempts were made to maintain the co-cultures for 15 days, but after 10 days, culture conditions started to deteriorate due to accumulation of cell debris and affected the concentration of both parasites and B-cells, and thus it was decided to limit the experiment to 10 days. During this time, RBCs were added to avoid overgrowth of parasites.

**Fig. 1:**
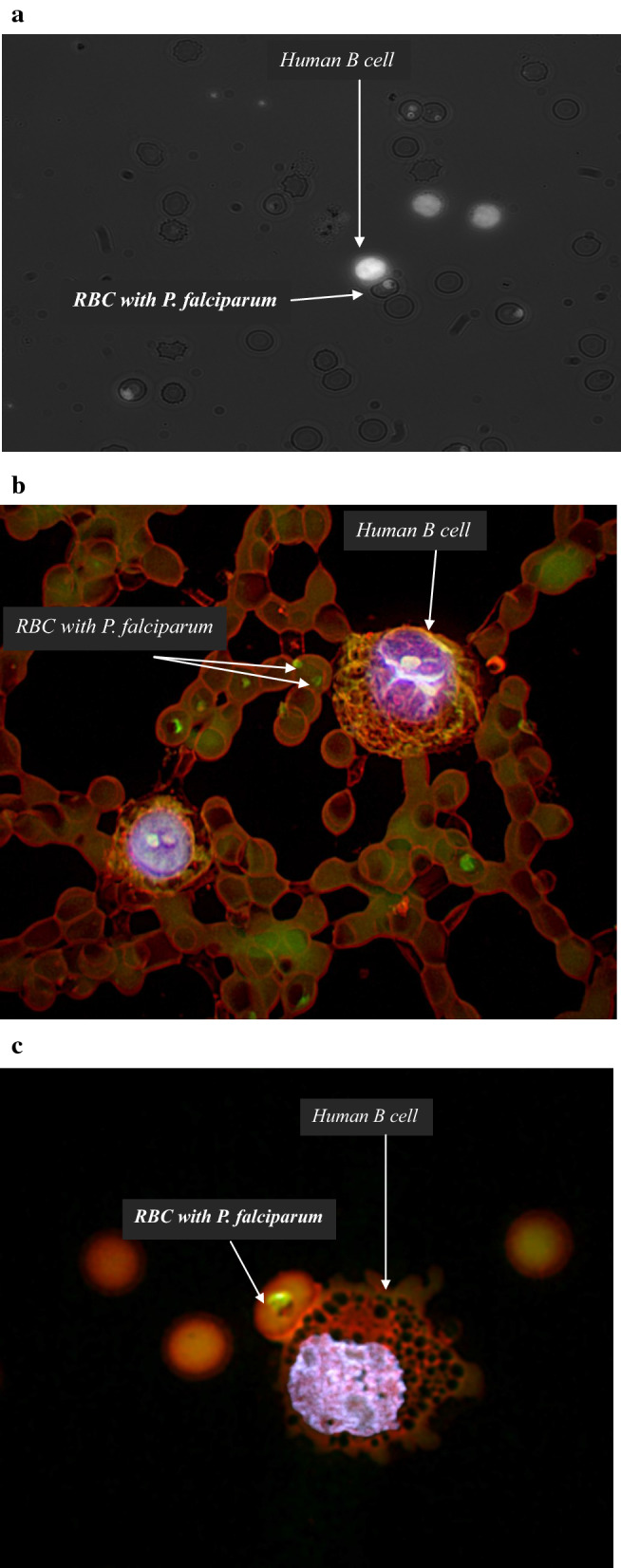
Co-culture of B-cells and *P. falciparum* (FCR3S1.2) followed by microscopy.** a** Acridine orange stain in light microscopy was used for daily monitoring. B-cells are seen as large fluorescent cells. *P. falciparum*-infected RBCs can be seen both in direct contact with a B-cell in the centre of the picture, and as separate cells. **b** Confocal microscopy. Numerous parasites can be seen inside RBCs. **c** Confocal microscopy showing a B-cell binding to a *P. falciparum*-infected RBC

**Fig. 2:**
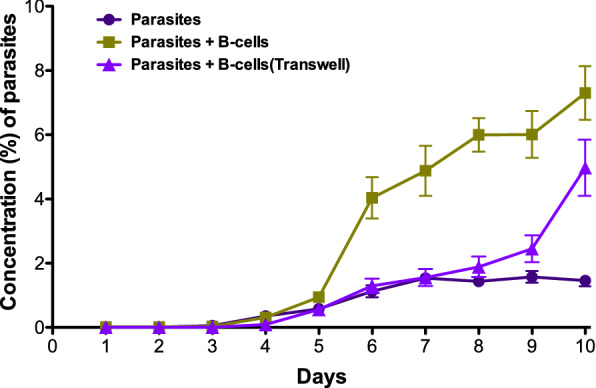
Development of parasite concentrations during 10 days of co-culture of FCR3S1.2 *Plasmodium falciparum* with and without human B-cells. When the parasite concentration exceeded 1–1.5% in at least one well, fresh RBCs were added equally to all wells. The mean results of eight experiments (four for Transwells) are shown, bars indicate SEM. Cultures were incubated in a 5% CO_2_ gas-flooded 37 °C incubator. Differences were significant at day 10 when Parasites were compared to Parasites + B-cells (p = 0.0006) and to Transwells (p = 0.001)

During the co-cultures, daily inspections were made microscopically (Fig. 1a, b). B-cells and infected RBCs were often in close contact (Fig. 1c). Parasites were only observed inside the RBCs, and never in the B-cells. The concentration of parasites consistently increased more rapidly and reached a higher peak in wells where B-cells were present (Fig. 2). These results clearly show that *P. falciparum* can multiply under the given co-culture conditions for 10 days and additionally display enhanced growth in the presence of B-cells. Similar results were achieved when FCR3S1.2 (a rosetting parasite line), 3D7 or FCR3S1.6 (both non-rosetting) were used.

To sustain the growth of both *P. falciparum* and B-cells in co-cultures, different proportions of 0–20% of human AB + serum and FBS were tested. The optimal mixture was 5% of each. More than 5% human serum resulted in excessive growth of parasites (culture crash) within a few days. Less than 5% human serum posed difficulties in sustaining parasite growth. IMDM was found to be superior to RPMI or DMEM for the growth of B-cells in the co-cultures.

**Fig. 3:**
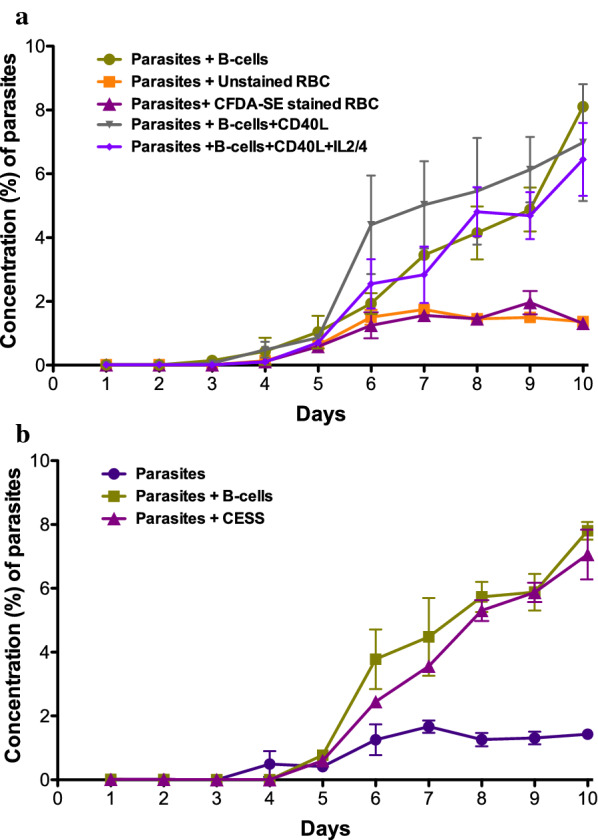
Co-cultures of B-cells and *P. falciparum*. **a** The addition of CD40L or IL2/4 or staining of RBCs with CFDA did not have any major impact on the percentage of parasites in the co-culture. When Parasites + Unstained RBC were compared to the other culture conditions, at day 10 there were significant differences for Parasites + B-cells (p < 0.0001), Parasites + B-cells + CD40L (p = 0.02) and Parasites + B-cells + CD40L + IL2/4 (p = 0.01). **b** The presence of CESS- or buffy-coat-derived B-cells led to a similar increase in the percentage of parasites in co-cultures (mean of 3 experiments). Error bars indicate SEM. When Parasites were compared to other culture conditions, at day 10 there were significant differences for Parasites + B-cells (p < 0.0001) and for Parasites + CESS (p = 0.002)

When culture conditions were finally optimized, the starting concentration of parasites was 0.01%, and the co-culture medium included recombinant CD40L, IL-4 and IL-2 with continuous addition of new RBCs to avoid overgrowth of parasites. The addition of CD40L or IL2/4 did not affect the percentage of parasites (Fig. 3a). Under these conditions, more than 90% of the B-cells were viable on day 10 (tested with trypan blue). Culture media was changed regularly (at least every second day) to replenish the nutrients required for the continuous cultures, and additional RBC were added to maintain the supply of fresh RBC to the parasites.

### The effect of direct contact between co-cultured cells

To investigate the effect of direct contact between B-cells and infected RBCs, the method was developed to include Transwell membranes. Firstly, 3 µm pore size membranes were tested but it was found that some RBCs passed through the filter. Instead, 0.4 µm pore size were used, which did not allow passage of cells or merozoites. Direct contact between cells resulted in increased numbers and faster growth of parasites (Fig. 1a), indicating that direct contact is advantageous for enhancing parasite growth.

### Increase in parasitaemia due to contact with B-cells

Parasitaemia was monitored daily by counting in a fluorescent microscope using acridine orange staining. Parasitaemia consistently increased more rapidly and peaked higher in the wells where iRBC were grown in direct contact with B-cells (Fig. 1). Fresh RBC were added uniformly to all wells when needed in order to keep the proliferation of parasites active. Towards the end of the 10 day co-culture, parasitaemia reached above 7% in wells containing B-cells in direct contact with iRBC. Parasitaemias in control wells with parasites alone peaked just under 2%. Parasitaemias in transwells increased and peaked at a level intermediate to the iRBC grown alone and the iRBC grown in direct contact with B-cells, indicating that direct contact is advantageous, but not necessary for increased growth of *P. falciparum*.

### Proliferation of B-cells in co-cultures

During the co-cultures, proliferation of B-cells was routinely observed, indicating that cells were viable and could sustain the co-culture conditions. When B-cells purified from human blood were used, there was a sevenfold increase in the number of cells on day 10 compared to day 0. With CESS there was a 15-fold increase. Control wells without parasites only showed a 1.2-fold increase for the B-cells from human blood, and a fivefold increase when CpG was added.

To exclude the possibility that the increase in human B-cells in the co-cultures was due to EBV, the culture medium alone (from an extra control well), B-cells only, and B-cells with parasites were tested for the presence of EBV using real-time PCR on day 10 of co-culturing. No EBV was detected in any of the tested cell cultures.

### CFDA-staining of RBCs

In order to evaluate if this new co-culture method could be utilized for future applications to study different populations of RBCs and to make real-time videos of live cultures, the suitability of CFDA stained RBCs was tested. The staining of RBCs did not have any effect on parasitaemia (Fig. 3a), indicating that CFDA staining is well suited for this type of experiment.

### Using CESS cell-line instead of B-cells from PBMC

In order to assess whether the increased parasitaemia observed in the co-cultures is not limited only to B-cells purified directly from human blood, the co-culture method was also developed to include a B-cell line (CESS). This resulted in a similar effect of increased growth of parasites (Fig. 3b) as when B-cells from PBMC were used.

### Visualization of B-cells and parasites in the co-culture

Culture components were witnessed directly under a light microscope (without staining) as well as after staining with acridine orange, Giemsa or OFB dye for confocal microscopy. All images confirmed the co-existence, survival and proliferation of both B-cells and parasites. For a more detailed depiction of the co-culture, confocal microscopy was used to visualize the co-culture at day 10 and the images clearly shows that there is direct contact between B-cells and malaria parasites.

### Analysis of B-cells using flow cytometry after 10 days of co-culture

**Fig. 4:**
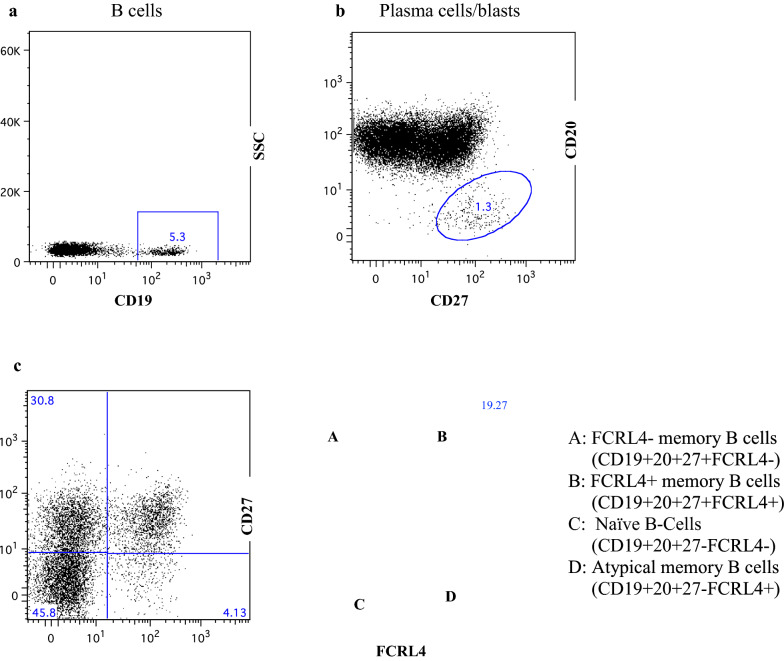
Representative flow cytometry plots to show gating strategy for phenotyping of B-cells. **a** Primary gating of CD19 + cells. Only these cells were considered for further analysis. **b** Gating of plasma cells/blasts. **c** Separation of CD27 ± and FCRL4 ± cells

**Fig. 5:**
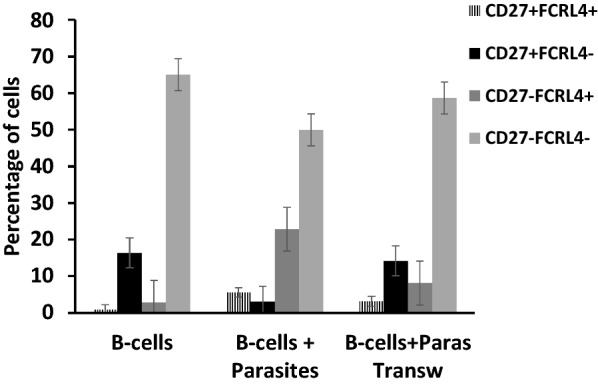
CD19 + CD20 + cells were measured using flow cytometry at day 10 of co-cultures. The proportion of atypical memory B-cells (CD27-FCRL4 +) cells was higher in the presence of parasites with a less prominent effect when Transwells were used. Mean of 3–5 experiments. Error bars show SEM. When all groups were compared to each other, there was a significant difference (p < 0.0001)

Growing *P. falciparum* and human B-cells together is potentially a good method for studying the expression of surface molecules on B-cells. At day 10, B-cells were analysed using flow cytometry. Representative plots for gating are shown in Fig. 4. For uniformity, all samples, with or without RBC, were treated with RBC lysis solution. Results for individual surface markers and marker combinations are shown in Fig. 5. B-cells that had been grown together with *P. falciparum* displayed an increase in the proportion of atypical memory B-cells (CD19 + CD20 + CD27 − FcRL4 +), especially when allowed direct contact (no Transwell). The proportion of classical memory B-cells (CD19 + CD20 + CD27 + FcRL4 −) contracted in presence of parasites, and also here a bigger effect was seen when direct contact was allowed.

### Analysis of immunoglobulins in co-cultures

During co-culturing, the addition of human serum was necessary to make the cultures sustainable, which caused a very high “background” level of immunoglobulins. Therefore, it was difficult to assess any production of immunoglobulins in the supernatants using ELISAs, especially for IgG, where no obvious differences could be observed between the wells. The production of IgM seemed to increase in the presence of parasites (0.1 increase in absorbance in ELISAs performed on the supernatant at the end of the co-culture period, mean of two different experiments), but since there was IgM added in the culture medium it was difficult to evaluate.

#### Human cytokine ELISA array

Of all the cytokines tested in the assay, only IL1α, IL-2, IL-4, IL-6, IL-8, GM-CSF and TNF-1β were detected above background the level. IL-2 and IL-4 both showed consistently high levels due to the addition in the culture medium. For the other cytokines, no obvious differences could be seen whether parasites were present or not.

## Discussion

In malaria endemic areas, most deaths in malaria occur during childhood, even though older individuals are exposed to new infections from mosquitoes all the time. This indicates that the parasites have developed a strategy to co-exist together with the human immune system for long periods of time. How this is achieved and how immunity against malaria is formed is still not known, although all parts of the immune system such as T-cells, B-cells and antibodies are important in the process [26–28]. Whole-blood assays have shown that even the uninfected erythrocytes inhibit *P. falciparum*-induced cellular immune responses through specifically inhibiting phagocytosis of infected red blood cells by peripheral blood monocytes, thus resulting in failure of antigen uptake and the lack of T-cell responses [29]. A basic requirement for progress in studies to formulate a vaccine, is available methods to perform suitable in vitro studies. In the past, B-cells and infected erythrocytes mixtures were incubated at 37 °C for 1 h only to visualize the binding and assess the secretory cytokines [30]. This paper shows that it is possible to successfully culture *P. falciparum* in 5% CO_2_ (in air). This should make parasite cultures easier to perform around the world since this type of incubator is often available in standard laboratories, and no special low-level oxygen gas is required. Only laboratory-adapted strains of *P. falciparum* (3D7, FCR3S1.2 and FCR3S1.6) have been tested in this paper, and only for 10 days, so it might be that fresh isolates directly obtained from patients still need a different environment for optimal survival, since they are often more challenging to keep in culture. For practical reasons though, most published in vitro studies of *P. falciparum* utilize laboratory-adapted strains.

The co-cultures were followed on a daily basis in order to ascertain the viability and morphology of both B-cells and parasites, and to observe direct contact between B-cells and iRBC, while manually counting the percentage parasitaemia. Formation of B-cell clusters in the co-culture indicated B-cell proliferation. Except for using B-cells purified from PBMC, CESS was also used, and both cells showed a similar increase in parasitaemia, indicating that it is really B-cells that is causing the increased growth of parasites (and not some other cell that might have been present in very small amounts).

With this novel method, there is an opportunity to perform studies of live parasites together with human B-cells in culture for 10 days, which is a major advance compared to previous attempts by others when co-cultures did not last for more than a couple of hours. This became possible because a culture medium was used together with a gas composition that was suitable for survival of both parasites and B-cells. Actually, one of the main problems in setting up this assay, was that when B-cells were added to the parasites, the growth (unexpectedly) increased so much that the cultures crashed just within a few days and fresh RBCs had to be added to keep parasitaemias down. When Transwells were used, no direct contact between the parasites and B-cells was possible and there was less growth of parasites. Towards the end of the 10 days though, there was an increase in parasitaemia even when Transwells were used, indicating that except for the advantageous effect of direct contact there is also some smaller component (such as cytokines for example) that can pass through the membranes and affect parasite growth.

Among the detected cytokines in the coculture supernatants, IL-1 is known to induce expression of pro-inflammatory cytokines and induction of fever. IL-4, IL-6 and IL-8 play key roles in induction of B-cell differentiation, antibody production and class switching. TNF-β induces the synthesis of GM-CSF, and mediates inflammatory and immune responses. IL-6 and IL-8 were detected by both ELISA and bead array methods, indicating that these cytokines are produced in the assay. Considering the key role, any disturbances to the levels of these cytokines could severely impact the B-cell growth and development. Apart from these, there could be some novel soluble factors secreted by parasites directly impacting the B-cell differentiation and memory B-cell development. Direct contact between cells might be more common than previously thought in vivo since RBCs infected with *P. falciparum* are often sequestered in small blood vessels, where the flow of blood is relatively slow, something that could facilitate direct interactions between cells. When parasites and B-cells were allowed to be in direct contact, there was an increase in the proportion of atypical memory B-cells and a decrease in classical memory B-cells, similar to what happens in patients in vivo. As this was less prominent when Transwells were used, it indicates that both soluble factors and more direct effects are responsible for the changes seen.

Both rosetting (FCR3S1.2) and non-rosetting (3D7 and FCR3S1.6) parasite lines were tested and there was no differences in the results, indicating that the ligands responsible for rosetting are not the ones of importance for interactions with B-cells. It has been shown before that CIDR1α (part of PfEMP1, expressed as a recombinant protein) is a T-cell independent polyclonal B-cell activator that binds to immunoglobulin [31], and attenuated IgG reactivity to PfEMP1 antigens is associated with Burkitt lymphoma [32]. When using this new co-culture assay, the number of FCRL4 + B-cells increased. It has been shown both in vitro and in controlled human *P. falciparum* infections that malaria affects development of memory B-cells [33, 34].

CpG is known to drive human transitional B-cells to terminal differentiation and production of natural IgM antibodies [26, 35], and was used to show that B-cells proliferated both in response to CpG and to parasites, but CpG did not lead to any changes in the measured memory B-cells, indicating that it is not a general stimulation that is the cause for the changes. In HIV, FCRL4 identifies a pro-inflammatory subset of B-cells [36]. In malaria it can be debated how functional the FCRL4 + (/or FRCL5 +) cells are compared to other B-cells [37], but since they occur in relatively large numbers in adults in malaria-endemic areas [38], they should be important to study to understand how immunity against malaria develops.

A limitation in extrapolating data from in vitro models to the in vivo situation, is of course that in the in vitro situation there is always a limited number of different cells, nutrients and reagents that can interact with each other. In this assay only B-cells and no other cells like neutrophils or endothelial cells for example were used, and these would also be present in vivo. However, if all the cells that are normally present in human blood were to be added in a single in vitro assay, it might be difficult to discern which cells that are responsible for a certain result. With this co-culture assay it is now at least possible for red blood cells containing live *P. falciparum* parasites and human B-cells to be maintained together. Since it is possible to maintain the cultures not only with a B-cell line but also with B-cells purified from human blood, this system could be adapted further for a wider range of other immune cells, and an advantage in this context is that it was possible to grow *P. falciparum* in 5% CO_2_, which is a gas that is often used for growth of different immune cells.

## Conclusions

In conclusion, *P. falciparum* parasites can be grown together with human B-cells and they can both affect each other. This method can be used to gain new information about interactions between different cells and parasite-infected RBCs. Multiplication of parasites increased substantially when B-cells were allowed to be in close contact, and an increase in atypical memory B-cells was seen when parasites were present. It is clear that both parasites and B-cells affect each other, and more studies are needed to understand this interaction in full, which should be helpful in understanding immunity against malaria and to develop a functional vaccine.

## Data Availability

All data generated or analysed during this study are included in this published article.

## Abbreviations

FCRL4: Fc receptor like 4
iRBC: Infected red blood cell
IMDM: Iscove’s modified Dulbecco’s medium
PBMC: Peripheral blood mononuclear cells
LCL: Lymphoblastoid cell line
FBS: Fetal bovine serum
DMEM: Dulbecco's modified Eagle’s medium
EBV: Epstein-Barr virus
CFDA-SE: Carboxyfluorescein diacetate succinimidyl ester
ELISA: Enzyme-linked immunosorbant assay
CIDR: Cysteine-rich interdomain region
PfEMP1: *Plasmodium falciparum* Erythrocyte Membrane Protein1
