# Publisher Correction to: Direct contact between *Plasmodium falciparum* and human B-cells in a novel co-culture increases parasite growth and affects B-cell growth

**DOI:** 10.1186/s12936-021-03853-5

**Published:** 2021-07-23

**Authors:** Sreenivasulu B. Reddy, Noemi Nagy, Caroline Rönnberg, Francesca Chiodi, Allan Lugaajju, Frank Heuts, Laszlo Szekely, Mats Wahlgren, Kristina E. M. Persson

**Affiliations:** 1 Microbiology, Tumor and Cell Biology, Karolinska Institutet, Stockholm, Sweden; 2 Department of Clinical Microbiology, Karolinska University Hospital, Huddinge, Stockholm, Sweden; 3 Makerere University, Kampala, Uganda; 4 Department of Laboratory Medicine, Skåne University Hospital, Lund University, Lund, Sweden

## Publisher Correction to: Malar J (2021) 20:303 10.1186/s12936-021-03831-x

Following publication of the original article [1], it was brought to our attention that the article had published with an incorrect version of Figure 4.

Figure 4 has since been updated in the published article and may be found in this correction for reference.

**Fig. 4:**
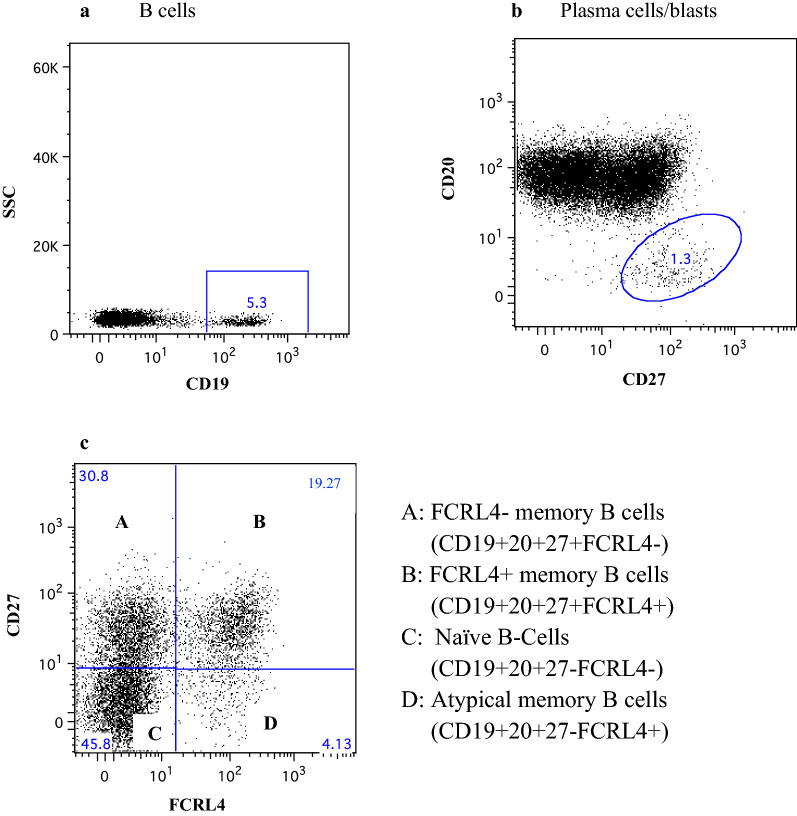
Representative flow cytometry plots to show gating strategy for phenotyping of B-cells. **a** Primary gating of CD19+ cells. Only these cells were considered for further analysis. **b** Gating of plasma cells/blasts. **c** Separation of CD27 and FCRL4 cells

The publisher apologizes for any inconvenience caused.
